# A Stroke With Pride and Prejudice: A Case Report

**DOI:** 10.7759/cureus.74584

**Published:** 2024-11-27

**Authors:** Samuel I Udo

**Affiliations:** 1 Emergency Medicine, Buckinghamshire Healthcare NHS Trust, Buckinghamshire, GBR

**Keywords:** carotid arteries, corpus striatum, dyspraxia, jane austen, magnetic resonance angiography, pride and prejudice, striatocapsular infarction syndrome, stroke, subcortical infarct

## Abstract

Dyspraxia is a brain pathology that affects an individual's movement, coordination, and motor skills. It makes everyday tasks, such as writing, balancing, or playing sports, more difficult due to problems with motor skills, planning, coordination, and balance. A form of dyspraxia that mainly affects the upper limbs has been recognised as a distinct clinical entity of subcortical strokes, classified as striatocapsular infarction syndrome (SIS). In this case report, a female writer presented with a vague history of right-sided headaches and sudden onset of difficulty with fine motor tasks. She brought in a paper with her typed summary from Jane Austen's Pride and Prejudice, which revealed the disorderliness in her writing and coordination. Examination subsequently revealed visual field defects and finger-nose dyspraxia. A CT head scan confirmed an established infarction in the right parietal lobe, consistent with stroke. Some patterns of these striatocapsular strokes may pose a challenge with early recognition and require comprehensive neurological examination, in addition to relevant neuroimaging for diagnosis. Prognosis varies and is not well defined, with thromboembolic infarction being a common denominator in complications.

## Introduction

The corpus striatum, a critical component of the basal ganglia, consists of the caudate nucleus, putamen, and ventral striatum (nucleus accumbens), which are subcortical structures primarily involved in movement control, habit formation, cognition, memory, and reward-based learning [1]. Located in the telencephalon, the corpus striatum receives input from the cortex and thalamus and projects to the globus pallidus, substantia nigra, and thalamus, forming complex circuits that regulate motor and cognitive functions [2]. The cerebral cortex, diencephalon, and brainstem are all intricately linked to these structures, which share a close anatomical and functional relationship [3]. Thus, lesions of the corpus striatum frequently cause a wide range of neurological dysfunctions because of this intricate circuitry.

Dyspraxia is a neurological pathology that affects how our brains and bodies work together, making everyday movements and actions a bit more challenging. People with dyspraxia often struggle with different things that seem like clockwork for others, such as writing, typing, or tying shoelaces; walking or running; planning and organising movements, like getting dressed or completing tasks; and coordination of various body parts, like catching a ball or riding a bike. For people with dyspraxia, it is not about being clumsy or careless; it is about having a brain that needs to work harder to connect the dots between thoughts and actions.

Jane Austen wrote Pride and Prejudice in 1813 [4]. The continued relevance of this book in the 21st century is demonstrated by its central role in facilitating a diagnosis of stroke in the emergency department.

This article was previously presented as a displayed poster for the 2023 Oxford School of Emergency Medicine (OSEM) Conference on 27th March 2023.

## Case presentation

A 51-year-old female writer walked into our Accident and Emergency Department with a nine-day history of right-sided headaches and a one-day history of difficulty coordinating routine hand tasks such as pouring coffee and typing.

The headache was of an insidious onset, felt as band-like waves, and was right-sided with a severity score of four out of 10. She believed the headaches were precipitated by recent stress, as she was intensively working on a huge ghostwriting project. She had relief with paracetamol. She had no associated symptoms like fever, vertigo, photophobia, rash, change in behaviour, nausea or vomiting, neck pain, blurring of vision, problems with sleep, nausea, or vomiting. She had never had any migraines in the past.

**Figure 1:**
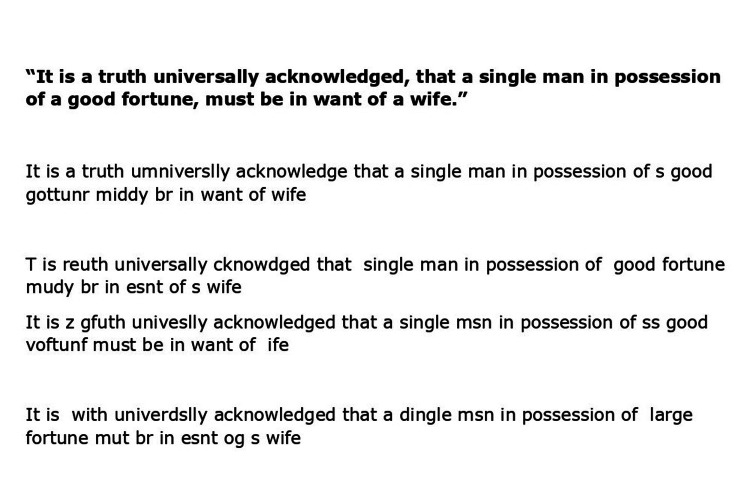
The patient's attempt to retype the first line from Jane Austen's Pride and Prejudice Original line in bold font. The patient's multiple attempts are beneath the bold font.

As she woke up and went to the kitchen on the morning of her hospital presentation, she found herself having trouble with certain things, such as putting milk into her coffee or flinging banana peels into the air instead of the waste basket. What disturbed her most, however, was her husband's emphasis on the gibberish she had just drafted after breakfast, which was supposed to be from the first lines of Jane Austen's Pride and Prejudice (Figure 1).

Her past medical history included type I diabetes mellitus (which was well-controlled with insulin), use of the progesterone intrauterine system (IUS) for contraception, and a significant smoking history of 20-pack years.

Examination on arrival in the emergency department revealed a Recognition of Stroke in the Emergency Room (ROSIER) score of 0. No cranial or gait abnormalities were identified. However, the loss of the right inferior nasal and left inferior temporal visual fields was noted, with left finger-nose dyspraxia.

Blood pressure was 132/67 mmHg, with an ECG showing normal sinus rhythm at a ventricular rate of 82 beats per minute. All other clinical parameters were within normal range. A diagnosis of suspected right hemispheric stroke was made based on the clinical history, writing sample, and examination findings. Her routine blood tests and ECG were unremarkable.

**Figure 2:**
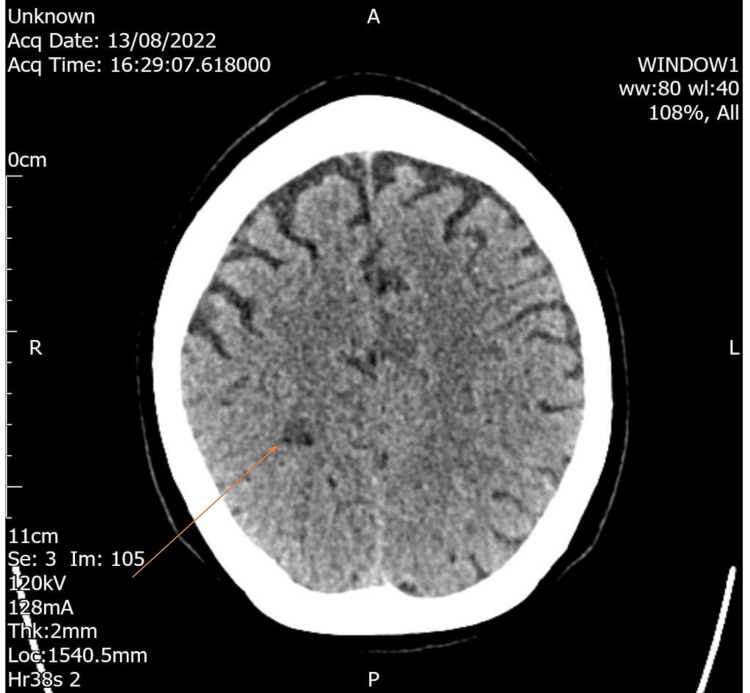
Focal area of hypoattenuation within the right parietal lobe (orange arrow), consistent with infarction

A computed tomography (CT) head scan performed within 30 minutes of presentation revealed a focal area of hypoattenuation within the right parietal lobe, which was consistent with established infarction (Figure 2).

There was no acute intra-axial haemorrhage, mass, or extra-axial collection. Alternatively, a normal configuration of the sulci and ventricles basal cisterns was present. There was no fracture or destructive osseous lesion. The patient was administered 300 mg aspirin and referred to the stroke team.

**Figure 3:**
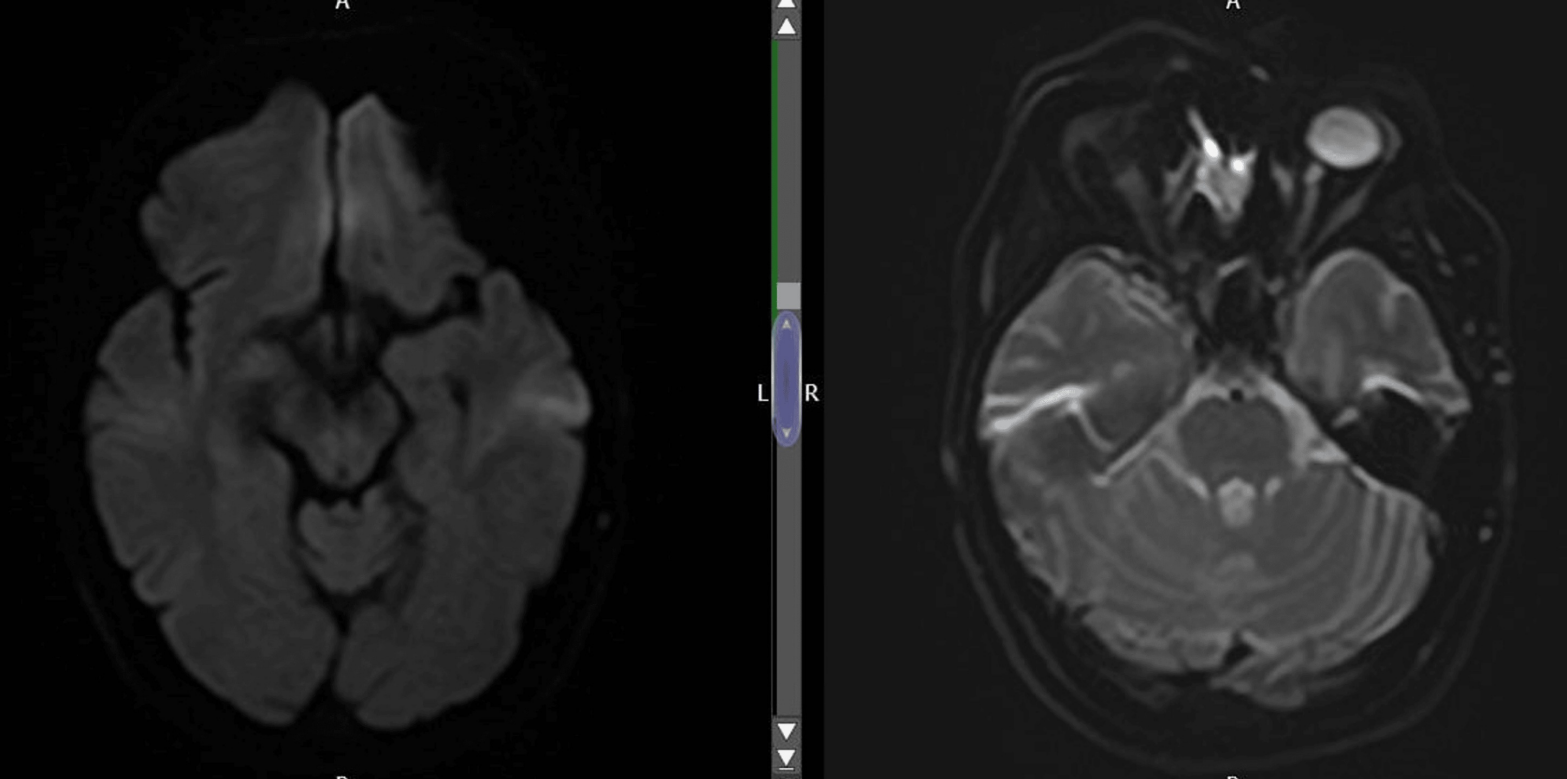
Multiple foci of diffusion restriction within the right internal and anterior watershed territories

Magnetic resonance angiography (MRA) of the carotids performed the next day revealed multiple foci of diffusion restriction within the right internal and anterior watershed territories and right parieto-occipital junction, in keeping with acute infarcts. The pattern of disease suggests a cardioembolic aetiology. No acute extra-axial collections were observed (Figure 3).

**Figure 4:**
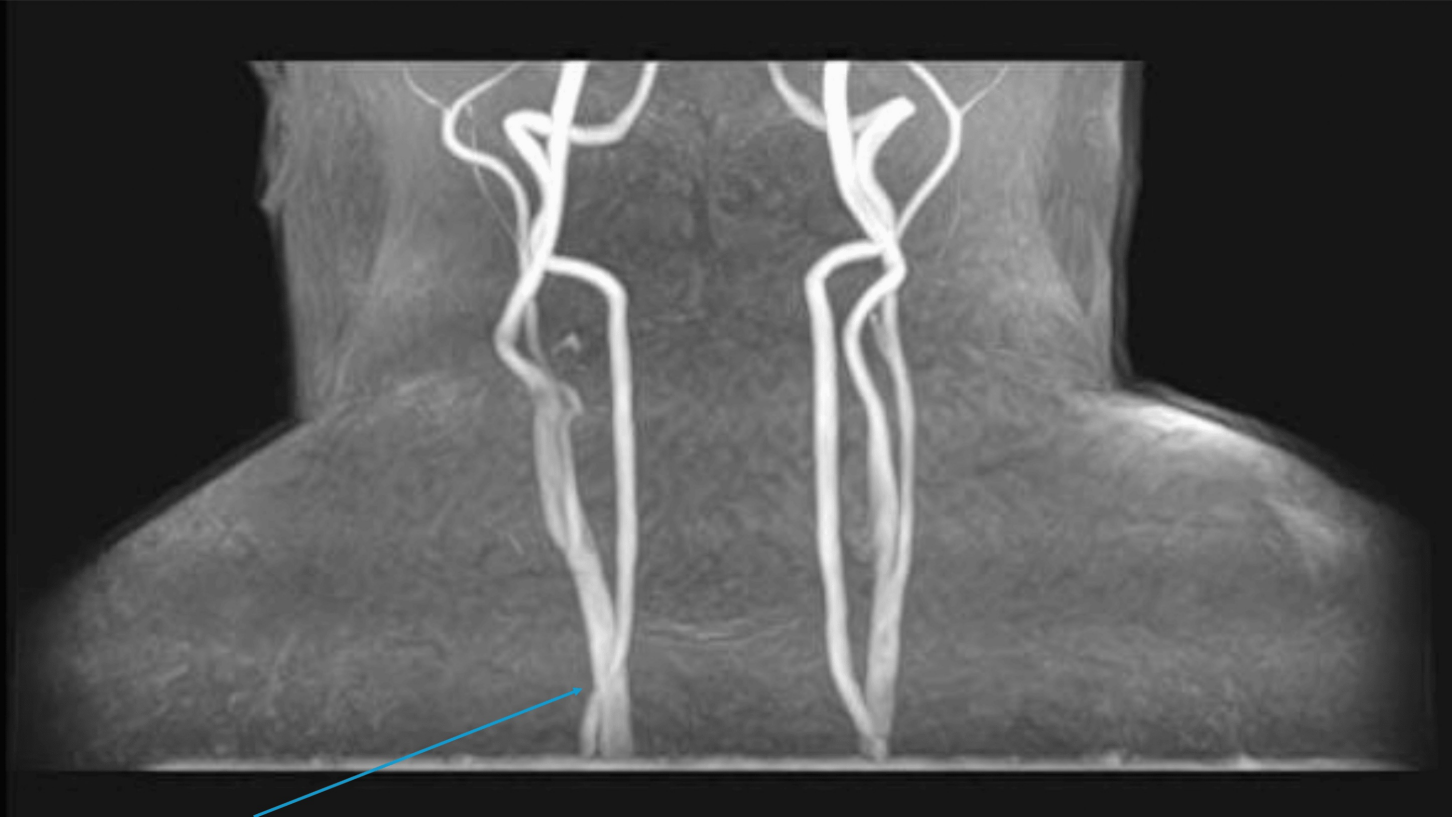
Magnetic resonance imaging of the carotids: radiological suggestion of significant eccentric short segment narrowing of the right internal carotid artery (blue arrow)

**Figure 5:**
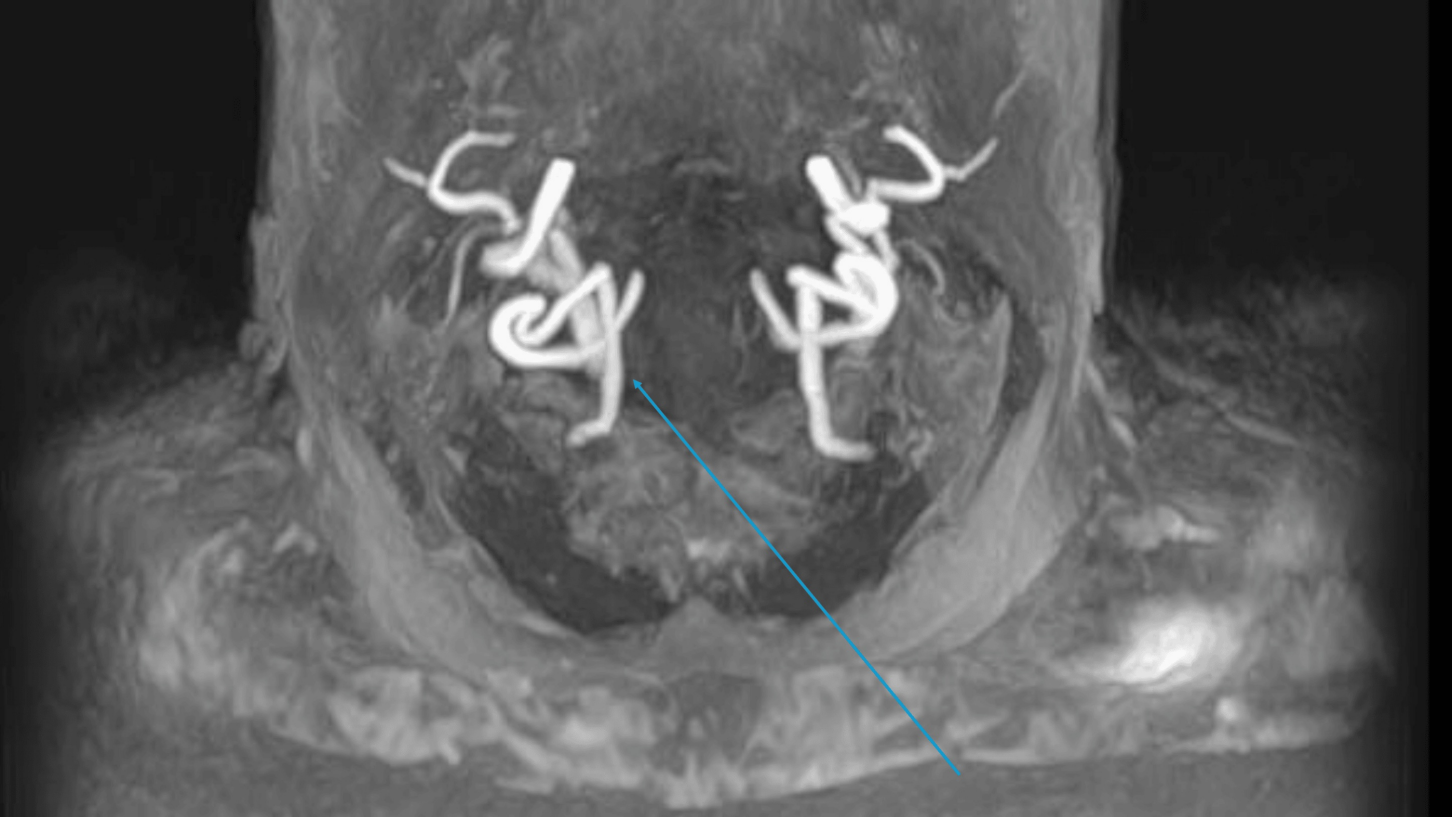
Magnetic resonance imaging of the carotids: eccentric short segment narrowing of the right internal carotid artery (blue arrow)

Magnetic resonance imaging of the carotids showed strong radiological suggestion of significant eccentric short segment narrowing of the right internal carotid artery, a short distance beyond its origin. Further clarification with ultrasound was suggested (Figures 4, 5).

**Figure 6:**
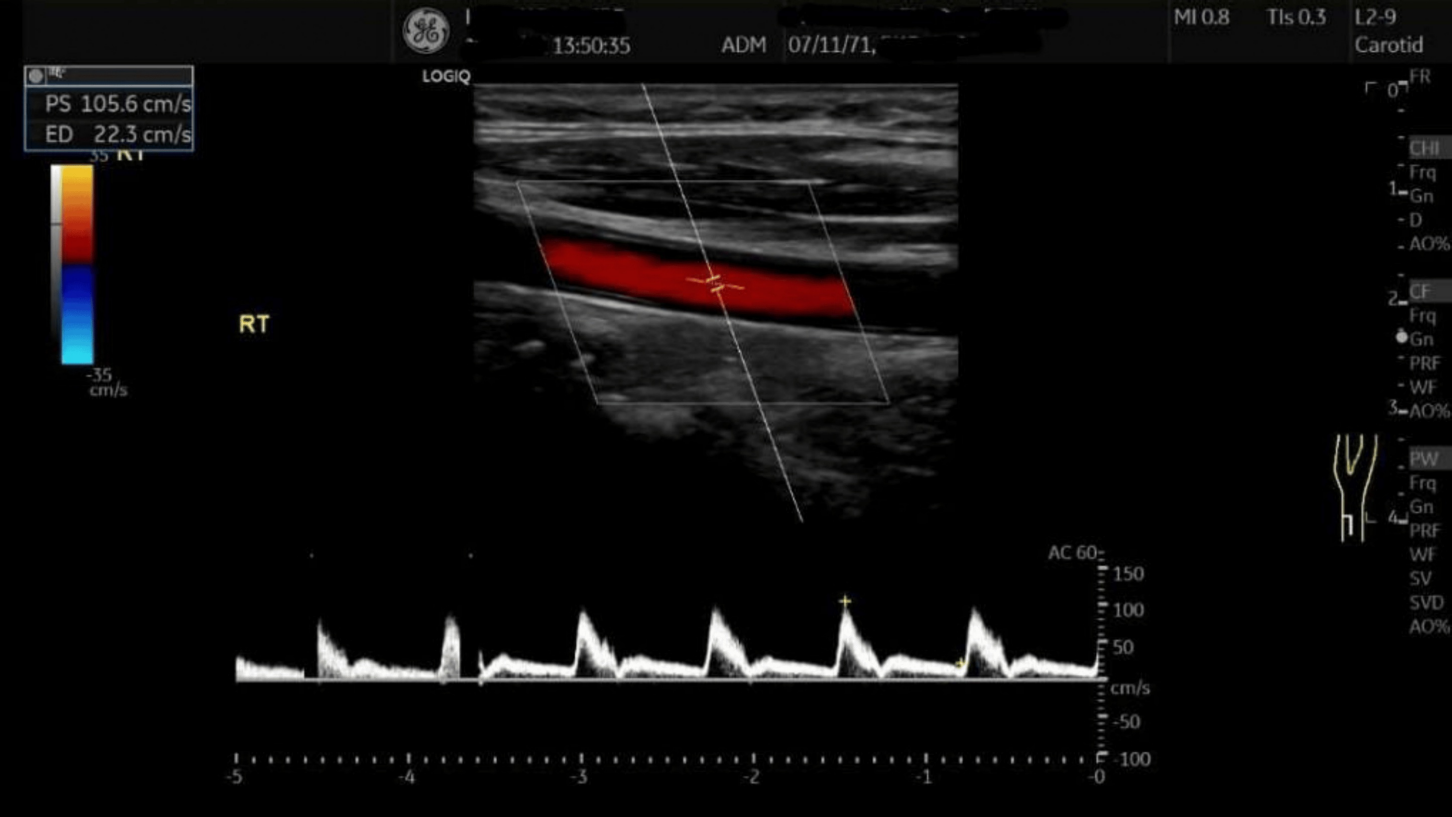
Doppler ultrasound of the carotids indicates normal findings

The Doppler ultrasound of the carotids was normal (Figure 6).

The patient was managed as a case of a watershed infarction by the stroke team, responded quite well to aspirin for two weeks and was switched to clopidogrel.

**Figure 7:**
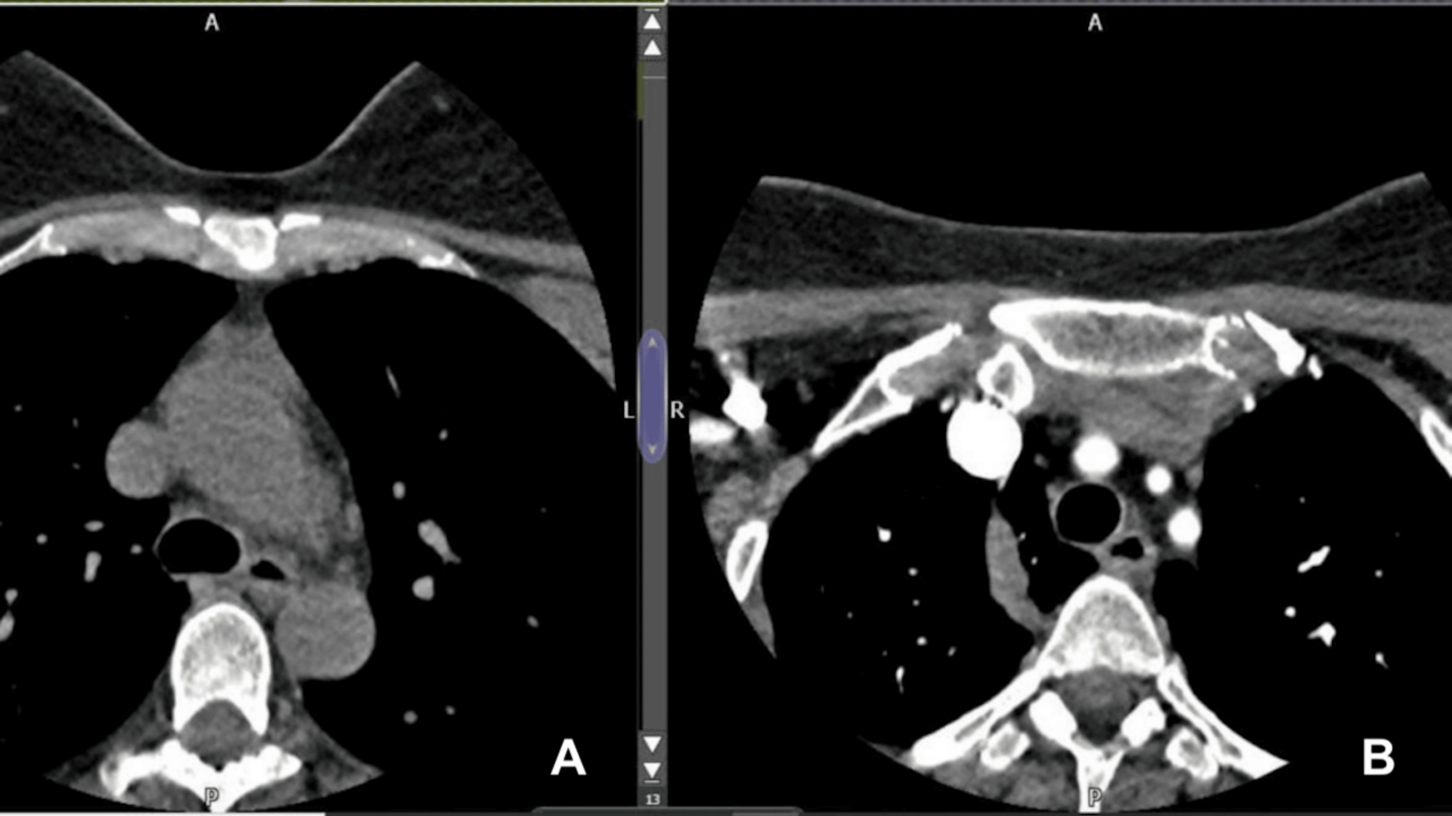
Computed tomography of the aortic arch and carotid angiogram indicate no significant stenosis (A) Pre- and (B) post-contrast images

**Figure 8:**
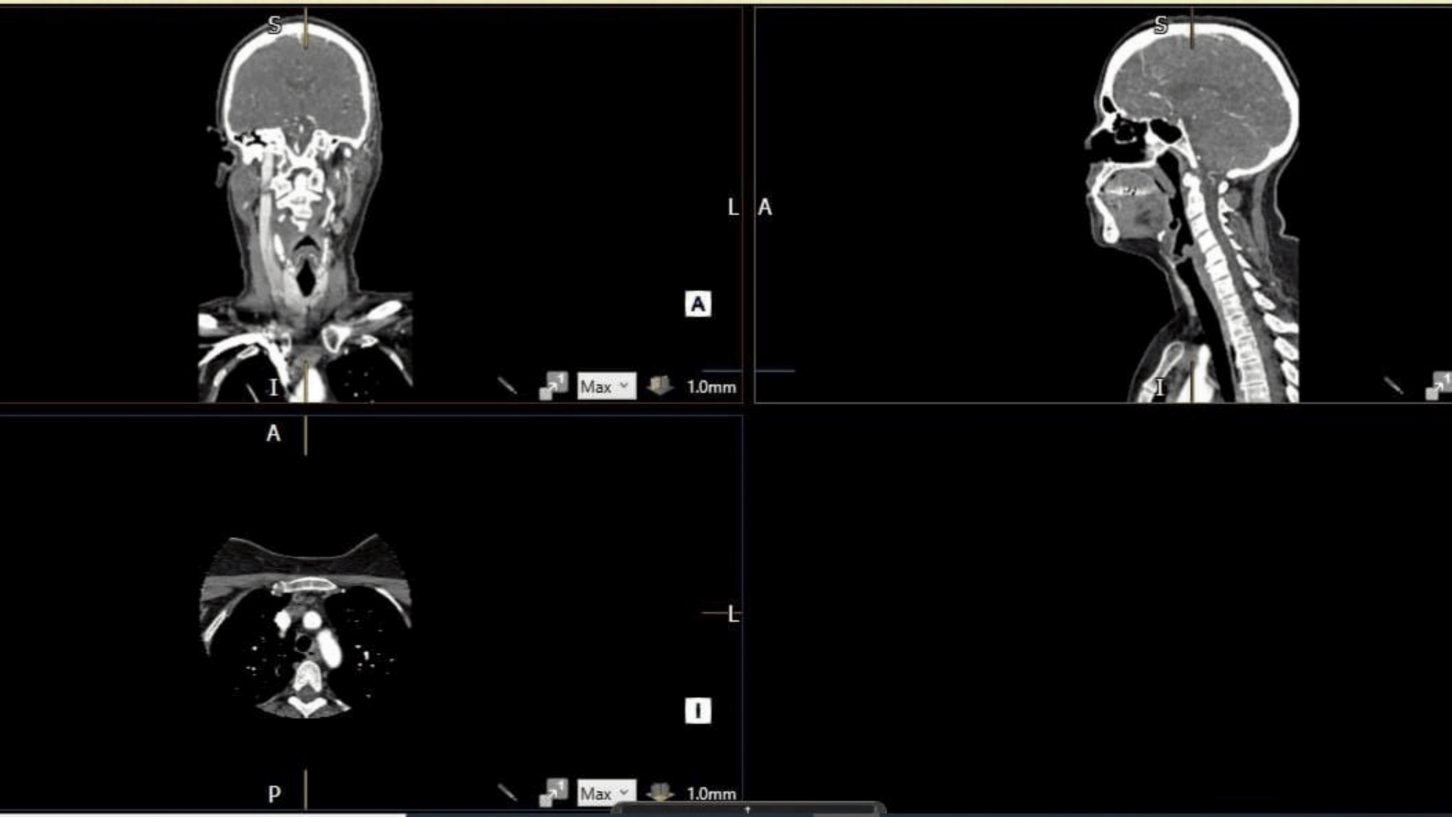
Computed tomography of the aortic arch and carotid angiogram indicate no significant stenosis 3D imaging

The CT of the aortic arch and carotid angiogram showed good opacification of the common carotid arteries, cervical internal carotid arteries, and vertebral and basilar arteries without evidence of significant stenosis. There was good opacification of the intracranial arteries without vascular abnormalities seen (Figures 7, 8).

She was referred for an outpatient cardiology follow-up, where no abnormalities and no evidence of paroxysmal atrial fibrillation or embolism were noted on the scheduled echocardiogram and transcranial Doppler ultrasound. She was subsequently referred to her family doctor (general practitioner) for targeted physiotherapy for her dyspraxia, with outpatient neurological follow-up, and is recovering very well.

## Discussion

This patient presented a new impairment evident in her writing paper sample due to a new circumscribed stroke lesion in her right parietal lobe, with the concomitant presence of other symptoms such as difficulty with other morning routine tasks and a loss of the right inferior nasal and left inferior temporal visual field loss with left finger-nose dyspraxia on examination. Screening tools such as the FAST score (face, arm, speech, and time of onset) and the ROSIER score recommended by the National Institute for Health and Care Excellence [5] (despite the absence of posterior circulatory lesions that would otherwise have made its use ambiguous) could not detect her stroke in this case.

Several studies have described a unique presentation of parietal lobe lesions called apraxic agraphia. The phrase "pure" apraxic agraphia was coined by Baxter and Warrington [6] to describe the writing disorder symptom that manifests without significant praxic or visual-constructional difficulties, as well as without any general language issues such as spelling or reading. Alexander et al. have also defined apractic agraphia as a writing impairment in which normal sensorimotor function, visual feedback, and word and letter knowledge are all present, but the actual orthographic production of letters and words is abnormal [7]. However, in this patient, her symptoms, although recognisable to the primary emergency physician through her typed words on paper, were consistent with medical text definitions of dyspraxia, which is overall poor performance of complex movements despite the ability to perform each individual component [8].

In a prospective study, 50 consecutive patients with striatocapsular infarction syndrome (SIS) were monitored for clinical and neuropsychological characteristics over a 10-year period. The pathogenesis and outcome were examined to provide insights into prognosis and management [9]. Among these patients, the most frequent clinical presentation was a stroke that primarily affected the upper limb and included cortical symptoms such as dyspraxia, neglect, or dysphasia. A study of risk factors and cerebral angiography was able to identify four pathophysiological subgroups: proximal middle cerebral artery abnormalities causing occlusion of multiple lateral striate arteries at their origins; severe extra-cranial cranial carotid artery occlusive disease with presumed embolism to the same site and/or involvement of haemodynamic factors; cardiac emboli to the origin of the middle cerebral artery; and normal angiography where pathogenesis was unknown [10].

When compared to age- and sex-matched controls with other types of ischaemic stroke, the risk factors for smoking and heart disease were elevated. At a mean follow-up of 2-25 years, the annual rate of stroke or vascular death was 2.7%. Little change in angiography, younger age, and only brachial or brachio-facial weakness with no cortical signs at presentation were predictors of a good recovery and return to a normal lifestyle [10].

In the spectrum of subcortical infarctions, this stroke entity merited recognition due to its unique pathogenesis, unique neuropsychological characteristics, and favourable prognosis [9,10-12]. Jane Austen's use of social commentary, realism, wit, and irony did not only earn her acclaim amongst critics and scholars, but her lines also helped define the severity and trajectory of clinical diagnosis of stroke, which presented in a prideful evasion of conventional stroke recognitions.

## Conclusions

Patients can perform normally on the rapid stroke assessment scales (e.g., the FAST and ROSIER scores) and still have a stroke. While this is challenging in a busy emergency department where diagnoses are based on rapid observations, the full clinical history, supported by an index of suspicion and a detailed neurological examination in the presence of a stroke risk factor, should become standard practice in all patients with unexplained neurology.

## Figures and Tables

**Figure 9:**
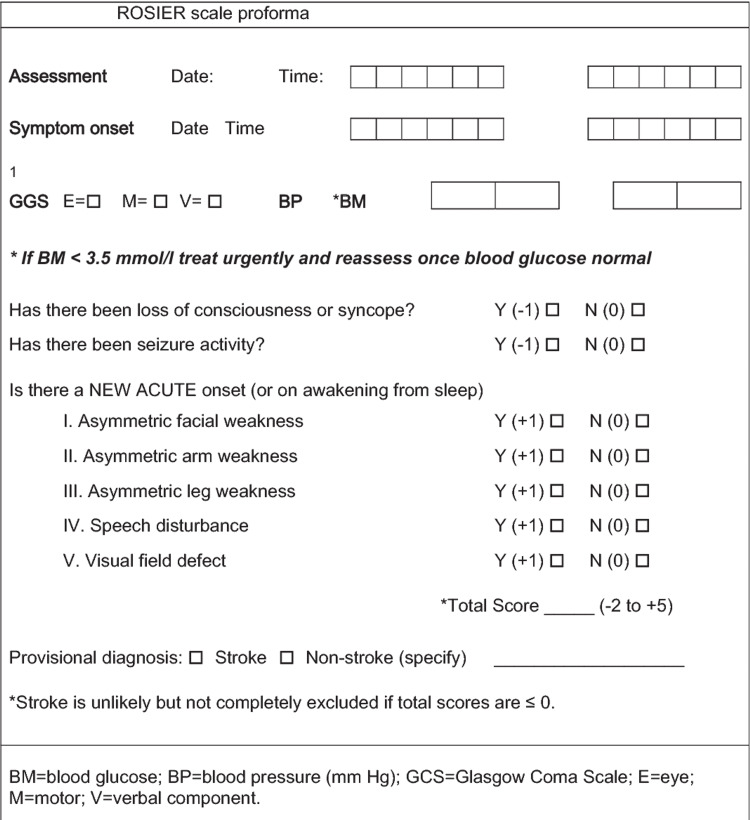
ROSIER scale ROSIER: Recognition of Stroke in the Emergency Room
